# Development of a Molecularly Evolved, Highly Sensitive CaMKII FRET Sensor with Improved Expression Pattern

**DOI:** 10.1371/journal.pone.0121109

**Published:** 2015-03-23

**Authors:** Akihiro C. E. Shibata, Hiroshi K. Maebashi, Yoshihisa Nakahata, Junichi Nabekura, Hideji Murakoshi

**Affiliations:** 1 Supportive Center for Brain Research, National Institute for Physiological Science, Okazaki, Aichi, Japan; 2 Division of Homeostatic Development, National Institute for Physiological Science, Okazaki, Aichi, Japan; 3 Department of Physiological Sciences, The Graduate University for Advanced Studies, Okazaki, Aichi, Japan; 4 Okazaki Institute for Integrative Bioscience, Okazaki, Aichi, Japan; 5 Core Research for Evolutional Science and Technology, Japan Science and Technology Agency (JST), Kawaguchi, Saitama, Japan; 6 Precursory Research for Embryonic Science and Technology, Japan Science and Technology Agency (JST), Kawaguchi, Saitama, Japan; UC San Diego, UNITED STATES

## Abstract

Genetically encoded fluorescence resonance energy transfer (FRET) biosensors have been successfully used to visualize protein activity in living cells. The sensitivity and accuracy of FRET measurements directly depend on biosensor folding efficiency, expression pattern, sensitivity, and dynamic range. Here, to improve the folding efficiency of the Ca^2+^/calmodulin-dependent protein kinase II alpha (CaMKIIα) FRET biosensor, we amplified the association domain of the *CaMKIIα* gene using error-prone polymerase chain reaction (PCR) and fused it to the N-terminus of mCherry in a bacterial expression vector. We also created an *Escherichia coli* expression library based on a previously reported fluorescent protein folding reporter method, and found a bright red fluorescent colony that contained the association domain with four mutations (F394L, I419V, A430T, and I434T). *In vitro* assays using the purified mutant protein confirmed improved folding kinetics of the downstream fluorescent protein, but not of the association domain itself. Furthermore, we introduced these mutations into the previously reported CaMKIIα FRET sensor and monitored its Ca^2+^/calmodulin-dependent activation in HeLa cells using 2-photon fluorescence lifetime imaging microscopy (2pFLIM), and found that the expression pattern and signal reproducibility of the mutant sensor were greatly improved without affecting the autophosphorylation function and incorporation into oligomeric CaMKIIα. We believe that our improved CaMKIIα FRET sensor would be useful in various types of cells and tissues, providing data with high accuracy and reproducibility. In addition, the method described here may also be applicable for improving the performance of all currently available FRET sensors.

## Introduction

Ca^2+^/calmodulin-dependent protein kinase II (CaMKII), a serine/threonine protein kinase, is abundantly expressed in hippocampal neurons [1,2] and is required for long-term potentiation (LTP) associated with learning and memory [3,4]. In the brain, CaMKII is present as a 12-subunit holoenzyme that is composed of α and β subunits at a ratio of 3:1 [5,6]. Binding of Ca^2+^/calmodulin to the CaMKII subunits induces conformational change [3,7], leading to an increase in kinase activity as well as subsequent autophosphorylation at the threonine residues (Thr286) in the regulatory regions of the adjacent subunits. Interestingly, even after calmodulin dissociation, CaMKII subunits continue to autonomously autophosphorylate each other, preventing dephosphorylation by phosphatases and allowing this protein to remain active [3]. Further, it has been hypothesized that this persistent CaMKII activation endures for many hours during LTP [8,9], and may function as a signaling molecule to maintain LTP [10].

For imaging CaMKII activity in living neurons, the fluorescent protein-based CaMKII fluorescence resonance energy transfer (FRET) sensor, Camuiα, was developed and has been successfully used to image the activity of this protein using ratiometric FRET measurement [11,12]. FRET is particularly useful in the detection of spatiotemporal protein activity, as it enables the measurement of the distance between two protein-bound fluorophores during protein–protein interactions or conformational changes [13,14]. Camuiα is a single-molecule FRET sensor, whereby two fluorescent proteins, a FRET donor and an acceptor, are fused to the N- and C-termini of CaMKIIα, respectively, to detect conformational changes. recently, Camuiα has been optimized for use with 2-photon fluorescence lifetime imaging microscopy (2pFLIM) [15,16] and utilized to monitor CaMKIIα activity in combination with 4-methoxy-7-nitroindolinyl-caged L-glutamate (MNI-caged glutamate) uncaging, which is used for inducing structural plasticity and LTP [16]. This analysis indicated that CaMKIIα activation is restricted to the stimulated spine and is only activated at the beginning of LTP [16].

In general, FRET measurements have yielded a multitude of useful data, and attempts to increase the sensitivity of the sensors have focused on improving the quality of fluorescent proteins [17–19] and optimization of the linkers [20]. However, improvements of the signaling protein itself have not been investigated. In this study, we attempted to improve a signaling protein region in the FRET sensor by applying the previously reported fluorescent protein folding reporter method in combination with error-prone polymerase chain reaction (PCR)-based random mutagenesis [21]. This method allowed us to improve the folding efficiency of the fluorescent protein fused to an association domain with four mutations. The improvement is likely due to the reduced interference of the mutant association domain with the fluorescent protein in the intermediate state. In addition, the introduction of four mutations into the Camuiα FRET sensor improved its expression pattern without affecting autophosphorylation. Furthermore, using 2pFLIM to monitor the FRET response in HeLa cells, we have shown that the use of the mutant Camuiα minimized the variability of the response signal after ionophore stimulation. Thus, this newly developed Camuiα mutant may be useful for the detection of CaMKIIα activity and we named it Camuiα4m. Moreover, the method described here may be generally applicable to other FRET sensors.

## Materials and Methods

### Random mutagenesis and screening

The rat *CaMKIIα* gene was used as the initial template for construction of the genetic libraries. Random mutagenesis was performed with error-prone PCR using the Diversity PCR random mutagenesis kit (Takara), whereby the association domain coding region of CaMKIIα (345–end) was amplified using Titanium Taq (Takara) according to the manufacturer’s instructions. Subsequently, PCR fragments were digested with *Eco*RI/*Not*I and ligated into the mCherry-inserted pRSET vector (Invitrogen), thereby fusing the amplified fragment to the N-terminus of the mCherry fluorophore with a linker sequence (GSGGRTS). In order to generate a library, this construct was then transformed into electrocompetent *Escherichia coli* DH5α cells and grown for 18–20 hours on ampicillin-containing LB/agar plates at 35°C. We then screened the mutants with improved folding using a blue-light transilluminator. The bright red fluorescent colonies were picked from the library and grown in LB media supplemented with antibiotics. The plasmids were then purified and sequenced. These steps were repeated a few times until four unique mutations were acquired.

### Plasmid construction

For construction of the Camuiα-mGsR and Camuiα-mGmC plasmids with mEGFP (monomeric enhanced Green Fluorescent Protein with the A206K mutation) and sREACh (super Resonance Energy-Accepting Chromoprotein) [22] or mCherry [23] pairs, the full-length *CaMKIIα* sequence was inserted into the modified pmEGFP-C1 with a linker coding the amino acid sequence SRLRSRA. Subsequently, sREACh or mCherry was subcloned into the C-terminal region of CaMKIIα with a linker coding the amino acid sequence GSGSGGSGGSG. For construction of the Camuiα mutants, a site-directed mutagenesis kit (Stratagene) was used. For construction of non-fluorescently labeled CaMKIIα mutants, calmodulin, and His-tagged association domain mutants, these DNA constructs were inserted into the modified pmEGFP-C1, replacing mEGFP. His-tagged association domain mutants for bacterial expression were constructed by inserting the domain mutants into a pRSET vector. For construction of the association domain mutants fused to super-folder GFP (sfGFP) [24], the association domain mutants were ligated with sfGFP into the pRSET vector, fusing the fragments to the N-terminus of sfGFP with the linker sequence GSG.

### Protein purification from bacteria and refolding kinetics

For the purification of the His-tagged association domain mutants and their sfGFP fusion, proteins were overexpressed in *E*. *coli* DH5α cells and purified with a Ni^+^-nitrilotriacetate (NTA) column (HiTrap, GE Healthcare). The concentration of the purified proteins was measured by absorption spectroscopy using the extinction coefficient of sfGFP (A_489_ = 83,000 cm^-1^·M^-1^) [24] or Bradford protein assay (Biorad).

To measure the refolding kinetics of sfGFP-fused association domains, proteins were denatured and refolded as described previously [25]. Briefly, the purified proteins (3–10 μM) were dissolved in denaturation buffer (8 M urea, 1 mM dithiothreitol (DTT)), and heated at 95°C for 5 minutes to denature both the association domain and sfGFP. The refolding reactions were initiated by diluting the denatured protein with a 100-fold amount of renaturation buffer (5 mM KCl, 2 mM MgCl_2_, 50 mM Tris pH 7.5, 1 mM DTT) at room temperature. The fluorescence recovery was measured using a fluorescence spectrophotometer at 510 nm (RF-5300PC; Shimadzu).

### Equilibrium unfolding measurement

To measure the tolerance of association domain for urea, His-tagged association domain mutants were purified and mixed with the various concentrations of urea in 25 mM HEPES, pH 7.3, containing 50 mM KCl and 5 mM DTT. The mixture was incubated overnight at 30°C. The samples were excited at 295 nm and the intrinsic tryptophan fluorescence spectra were recorded from 310 to 450 nm using a fluorescence spectrophotometer as described elsewhere [26].

### Circular dichroism (CD) spectroscopy

To measure the CD spectra after refolding of association domain mutants, 20 μM of proteins were denatured with 8 M urea and 1 mM DTT, and were incubated for 1 hour at 37°C. The refolding reactions were initiated by diluting the denatured protein with a 5-fold dilution at room temperature. After 3 minutes of initiation, the CD measurement was carried out. CD spectra were recorded using a Jasco J-720WI spectropolarimeter (JASCO) at room temperature. All samples containing 4 μM of protein were measured in a buffer (20 mM phosphate, pH 7.3, 50 mM KCl) with 1-mm path lengths. The baseline spectra of buffer and denaturant without protein were subtracted from respective protein spectra, as described previously [26].

### HeLa cell preparation

HeLa cells were cultured in Ham’s F12 medium supplemented with 10% fetal bovine serum at 37°C in 5% CO_2_ and transfected with the plasmids using Lipofectamine 2000 (Invitrogen) followed by a 18–24 hour incubation. Epifluorescence imaging and 2pFLIM observation was performed in a solution containing HEPES (30 mM, pH 7.3)-buffered artificial cerebrospinal fluid (ACSF) (130 mM NaCl, 2.5 mM KCl, 1 mM CaCl_2_, 1 mM MgCl_2_, 1.25 mM NaH_2_PO_4_, and 25 mM glucose) at room temperature. For ionophore stimulation, 4-bromo-A23187 ionophore (Sigma) was added at the indicated concentration.

### Dissociated culture of hippocampal neurons and transfection

Cultured hippocampal neurons were prepared as described elsewhere [27] with minor modifications. Briefly, postnatal day 1 rats were anesthetized and decapitated, followed by brain removal and hippocampal tissue dissection. Neurons triturated by papain treatment were plated at a density of 1.3 × 10^4^ cells/cm^2^ on polyethyleneimine-coated 35 mm culture dishes and maintained in serum-free Neurobasal medium supplemented with 2% B27, 2 mM GlutaMAX-I, and 10 mM HEPES at 37°C and 5% CO_2_. After 12 days, neurons were transfected with 2 μg of either Camuiα-mGsR or Camuiα4m-mGsR plasmid using Lipofectamine 2000 (Invitrogen) according to the manufacture’s protocol. After 18–24 hours, the neurons in the buffer (10 mM HEPES, 150 mM NaCl, 2.5 mM KCl, 2 mM CaCl_2_, 1 mM MgCl_2_, 10 mM glucose, and Tris-base for adjusting to pH 7.4) were imaged under the epifluorescence microscope.

### Hippocampal slice culture and gene gun transfection

Hippocampal slice culture was prepared from postnatal day 6–7 rats, as described previously [28] with minor modifications. In brief, pups were anesthetized and decapitated, and subsequently the brains were taken out. The hippocampus was taken out from a brain and cut with a tissue chopper (McIlwain) at the 350 μm thickness in ice-cold dissection medium (25 mM HEPES, 2 mM NaHCO_3_, 4 mM KCl, 5 mM MgCl_2_, 1 mM CaCl_2_, 10 mM D-glucose, 248 mM sucrose). The slices were cultured on the membrane inserts (PICM0RG50, Millipore) placed on culture media (50% MEM, 21% HBSS, 15 mM NaHCO_3_, 6.25 mM HEPES, 10 mM D-glucose, 1 mM L-glutamine, 0.88 mM ascorbic acid, 1 mg/mL insulin, 25% horse serum), and incubated at 35°C in 5% CO_2_. After 7–12 days in culture, CA1 pyramidal neurons were transfected with a gene gun (Ningbo Scientz Biotechnology) using 1.6 μm gold particles coated with plasmids, and imaged after 2–5 days. For making bullets, the amount of gold particles and DNAs used for 30 cm tubes were 16 μg Camuiα-mGsR + 6 mg gold for spine counting experiments; 16 μg Camuiα-mGsR + 10 μg tandem mCherry [29] + 6 mg gold for single spine glutamate uncaging experiments.

### Epifluorescence imaging

HeLa cells or dissociated neurons expressing Camuiα mutants were excited by passing a blue light (475 nm LED; CoolLED) through an excitation filter (FF01-510/84; Semrock). The fluorescence images were taken with a CCD camera (DP80; Olympus) or EM-CCD camera (ImagEM9100-13; Hamamatsu Photonics) mounted on a microscope (BX51WI; Olympus) through a 40× or 60× objective lens and an emission filter (FF01-525/39; Semrock).

### Two-photon fluorescence lifetime imaging and glutamate uncaging

Details of FLIM-FRET imaging using a custom-built 2pFLIM have been described previously [30]. Briefly, the mEGFP in Camuiα mutant was excited with a Ti-sapphire laser (Mai Tai; Spectra-Physics) tuned to 920 nm for HeLa cells and 960 nm for neurons, and was visualized through an objective lens (60×, 0.9 NA; Olympus). The scanning mirror (6210H; Cambridge Technology) was controlled with a PCI board (PCI-6110; National Instruments) and ScanImage software [31]. The fluorescence photon signals were collected by a photomultiplier tube (H7422-40p; Hamamatsu) placed after the emission filter (FF01-510/84; Semrock). Fluorescence lifetime measurement was carried out using a time-correlated single photon counting board (SPC-150; Becker & Hickl) controlled with custom software [30]. For construction of the fluorescence lifetime image, the mean fluorescence lifetime at each pixel was translated to a color-coded image [32]. Analysis of the lifetime change for individual cells was carried out as described previously [16].

Two-photon glutamate uncaging was carried out in Mg^2+^-free HEPES-buffered ACSF (10 mM HEPES, pH 7.4, 135 mM NaCl, 2.5 mM KCl, 4 mM CaCl_2_, 1.25 mM NaH_2_PO_4_, and 12.5 mM glucose, 1 μM tetrodotoxin (TTX), 2 mM MNI-glutamate) at 24–26C. Single-spine structural plasticity was induced with a train of 6 ms uncaging pulses (30 times at 0.5 Hz) using a Ti-sapphire laser tuned to 720 nm at 8 mW laser power under the objective lens.

### Protein purification from HeLa cell and Native-PAGE

For the purification of the His-tagged association domain mutants from HeLa cells, cells were transfected with the plasmids using Lipofectamine 2000 followed by a 24-hour incubation. The cells were lysed in lysis buffer (1% Triton X-100, 50 mM Tris-HCl, pH 7.5, 150 mM NaCl, 10 mM MgCl_2_, 4 mM EDTA, 5% glycerol, 5 mM imidazole) and centrifuged. Supernatants were incubated with resin (Ni-Sepharose 6 Fast Flow; GE Healthcare) for 1 hour. Samples were washed three times with 25 mM imidazole and two times with 50 mM imidazole in wash buffer (25 mM HEPES, pH7.3, 50 mM KCl). Subsequently, proteins were released from resin using 500 mM imidazole in wash buffer. For native-PAGE, the purified proteins were incubated in a buffer (25 mM HEPES, pH7.3, 50 mM KCl, 1 mM DTT) for 1 hour at 37°C and mixed with 4 × sample buffer (0.2 M Tris, pH6.8, 40% glycerol, 2.5% bromophenol blue). Electrophoresis was carried out as described previously [33], and silver staining was performed using silver stain reagent kit (Cosmo Bio).

### Immunoprecipitation and kinase assays

For the immunoprecipitation experiments, Camuiα-mGsR or Camuiα4m-mGsR was co-transfected with CaMKIIα or CaMKIIα4m into HeLa cells. After 18–24 hours, the cells were lysed in lysis buffer (1% Triton X-100, 50 mM Tris-HCl, pH 7.5, 150 mM NaCl, 10 mM MgCl_2_, 4 mM EDTA, 5% glycerol) and centrifuged. Supernatants were incubated with anti-GFP mouse monoclonal antibody (598; MBL) for 3 hours and then incubated with protein G beads for 1 hour at 4°C. Samples were washed three times with wash buffer (20 mM Tris-HCl, pH 7.5, 150 mM NaCl, 2 mM MgCl_2_) and re-dissolved in SDS sample buffer. Then, western blotting was performed with anti-CaMKII antibody (#4436S; CST) and goat-anti-rabbit-HRP (Jackson).

For the kinase assays, Camuiα-mGsR or Camuiα4m-mGsR was co-transfected with calmodulin (1:1) into HeLa cells. After 18–24 hours, the cells were stimulated with 20 μM ionophore for 1 or 2 minutes at 30°C, lysed in lysis buffer with 1% NP-40 instead of 1% Triton X-100 in the presence of phosphatase inhibitor cocktail tablets (PhosphoSTOP; Roche), and centrifuged. Supernatants were re-dissolved in SDS sample buffer and analyzed by western blotting using anti-phosphoThr286 CaMKII antibody (#3361S; CST) and goat-anti-rabbit-HRP (Jackson).

## Results and Discussion

**Fig 1:**
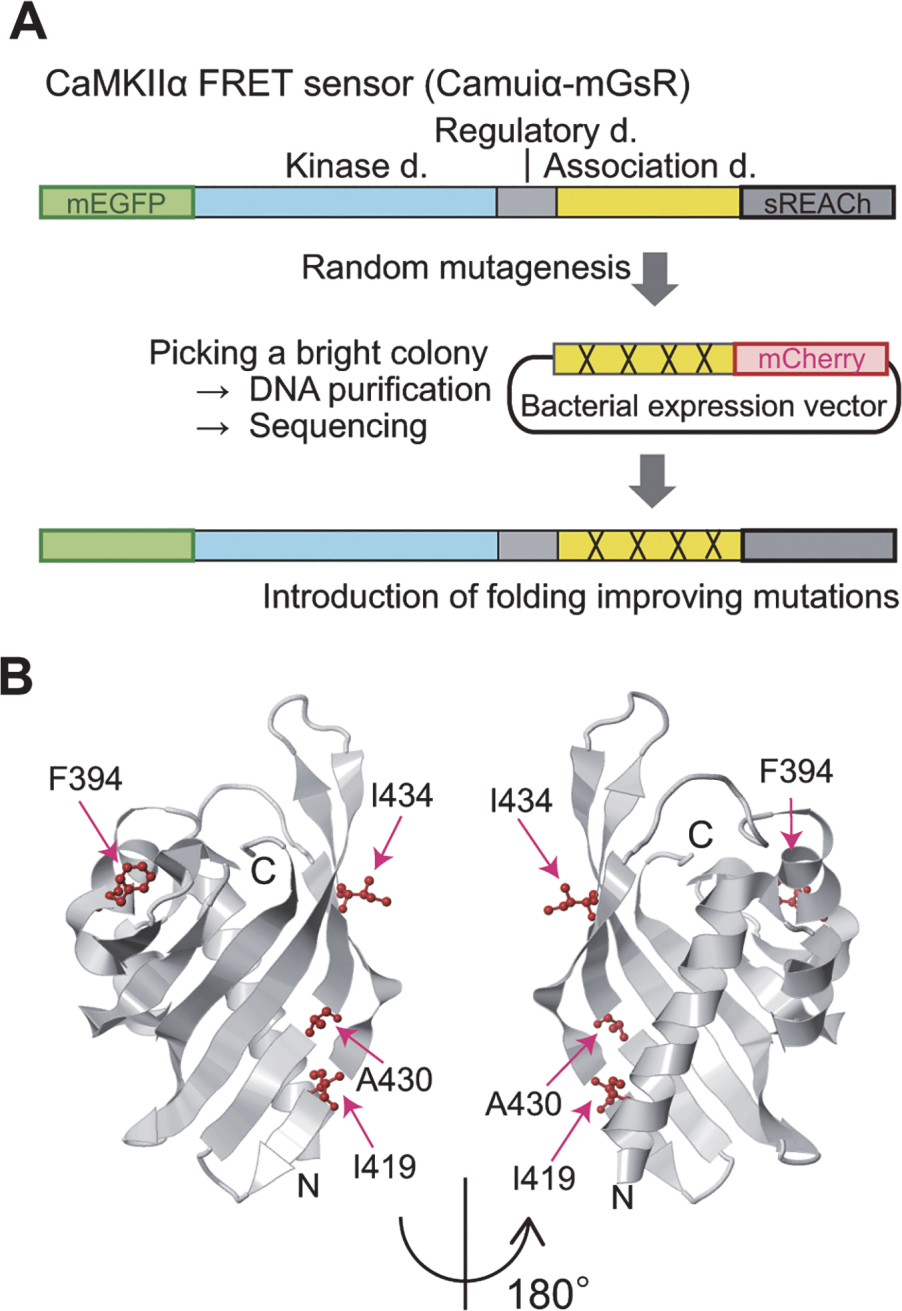
Schematic diagram of CaMKIIα FRET sensor evolution. **(A)** The CaMKIIα fluorescence resonance energy transfer (FRET) sensor (Camuiα) consists of FRET donor (mEGFP) and acceptor fluorophores (sREACh) and full-length CaMKIIα, which contains kinase, regulatory, and association domains. For improving the folding efficiency of the sensor, the association domain was amplified by error-prone PCR and subcloned into a bacterial expression vector. Subsequently, the ligated DNA was transformed in *Escherichia coli* to make a library. Bright red colonies were isolated from the library and analyzed by DNA sequencing. The identified mutations were then incorporated into the FRET sensor, leading to an increase in its folding efficiency. This scheme is based on an assumption that the folding efficiency of the association domain is coupled to that of mCherry. **(B)** The crystal structure of the association domain of human CaMKIIβ7 (PDB code: 3SOA). Note that this sequence is identical to the corresponding region in rat CaMKIIα (345–474) used in this study. The positions of four mutations (F394L, I419V, A430T, and I434T) found in this study are shown in red.

To improve the folding efficiency of the previously reported Camuiα [11,16], we decided to improve a domain of CaMKIIα, rather than the full-length protein, since CaMKIIα is over 50 kDa and is difficult to express in *E*. *coli* in general. CaMKIIα consists of a kinase domain and an association domain, and since the introduction of mutations in the kinase domain may change the enzymatic specificity of the protein, we focused on the association domain (Fig. 1A). We used a modified version of the GFP folding reporter method in combination with error-prone PCR [21] to increase folding efficiency. As a reporter fluorescent protein, we used mCherry instead of GFP, since the chromophore maturation time of mCherry is faster [23,34], which facilitates the screening in a short time. The PCR products coding the association domain with random mutations were then fused to the N-terminus of mCherry and ligated into a bacterial expression vector, constructing a library (Fig. 1A). After screening more than 50,000 transformed colonies, we found several bright red colonies. After multiple iterations of these experiments, we found an extremely bright red colony, and the subsequent sequence analysis showed four substitutions, F394L, I419V, A430T, and I434T in the association domain (Fig. 1B), but no mutations in mCherry. Since this method is based on the assumption that the productive folding of fluorescent protein is related to the characteristics of the upstream protein [21], the bright red colonies probably indicate the decreased chance for entanglement and aggregation between the upstream and downstream mCherry proteins [35].

**Fig 2:**
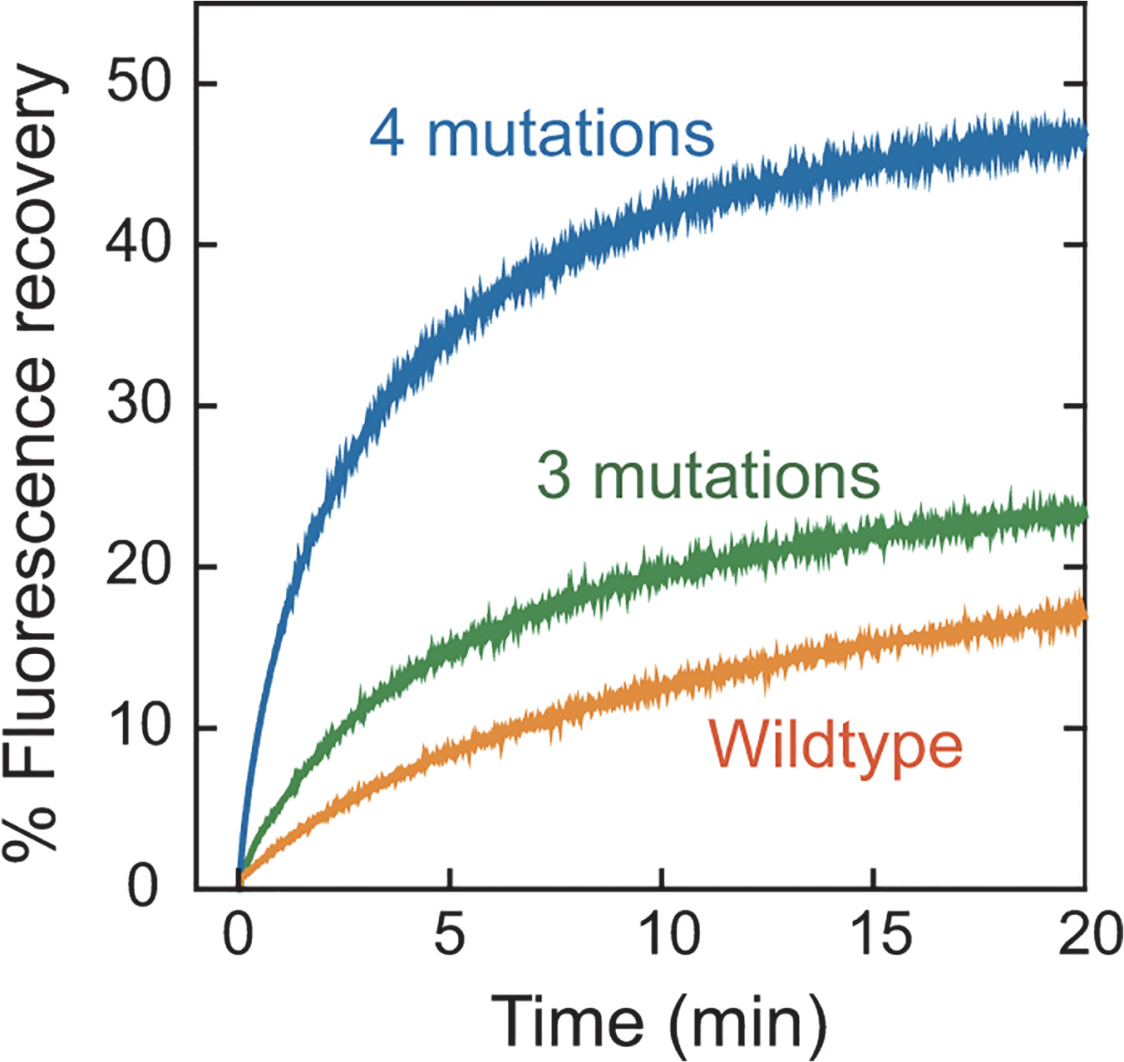
Comparative characterization of the folding properties of sfGFP fused to the association domain mutants. Refolding of the denatured sfGFP in the association domain mutants was initiated by a 100-fold dilution with renaturation buffer at room temperature. Fluorescence recovery of sfGFP in the association domain mutants was monitored by a fluorescence spectrometer (excitation at 488 nm, emission at 510 nm). Wild-type (no mutations), orange; 3 mutations (F394L, A430T, and I434T), green; 4 mutations (F394L, I419V, A430T, and I434T), blue.

To evaluate the effects of the individual mutations in the association domain on folding kinetics of the downstream fluorescent protein, we fused the respective mutants to the N-terminus of sfGFP and purified them using conventional His-tagged protein purification. The purified proteins were then denatured in urea [25], and the recovery of sfGFP fluorescence upon refolding treatment was monitored [21] (Fig. 2, Table 1). The fluorescence of the denatured wild-type association domain fused to sfGFP only recovered up to 21% with a half-time of 7 minutes. Similarly, the mutant containing three mutations (F394L, A430T, and I434T) showed only 25% recovery, but had a 2-fold improved half-time (3.5 minutes). In contrast to these results, the mutant containing four mutations exhibited a 2-fold higher recovery (46%) and a 4-fold faster half-time (about 2 minutes) compared with the wild-type, clearly indicating improved folding efficiency of the sfGFP (Fig. 2, Table 1).

**Table 1:** Folding parameters of the association domain (AD)-sfGFP mutants.

AD mutant	T_1/2_ (sec)	% Recovery
sfGFP only	14	70
WT	424	21
F394L	411	26
I419V	299	14
A430T	331	22
I434T	348	30
F394L/I434T	351	32
A430T/I434T	275	30
F394L/A430T/I434T	209	25
F394L/I419V/A430T/I434T	118	46

T_1/2_: Half-time for sfGFP recovery

% Recovery at ∞ minutes was estimated by exponential fitting

**Fig 3:**
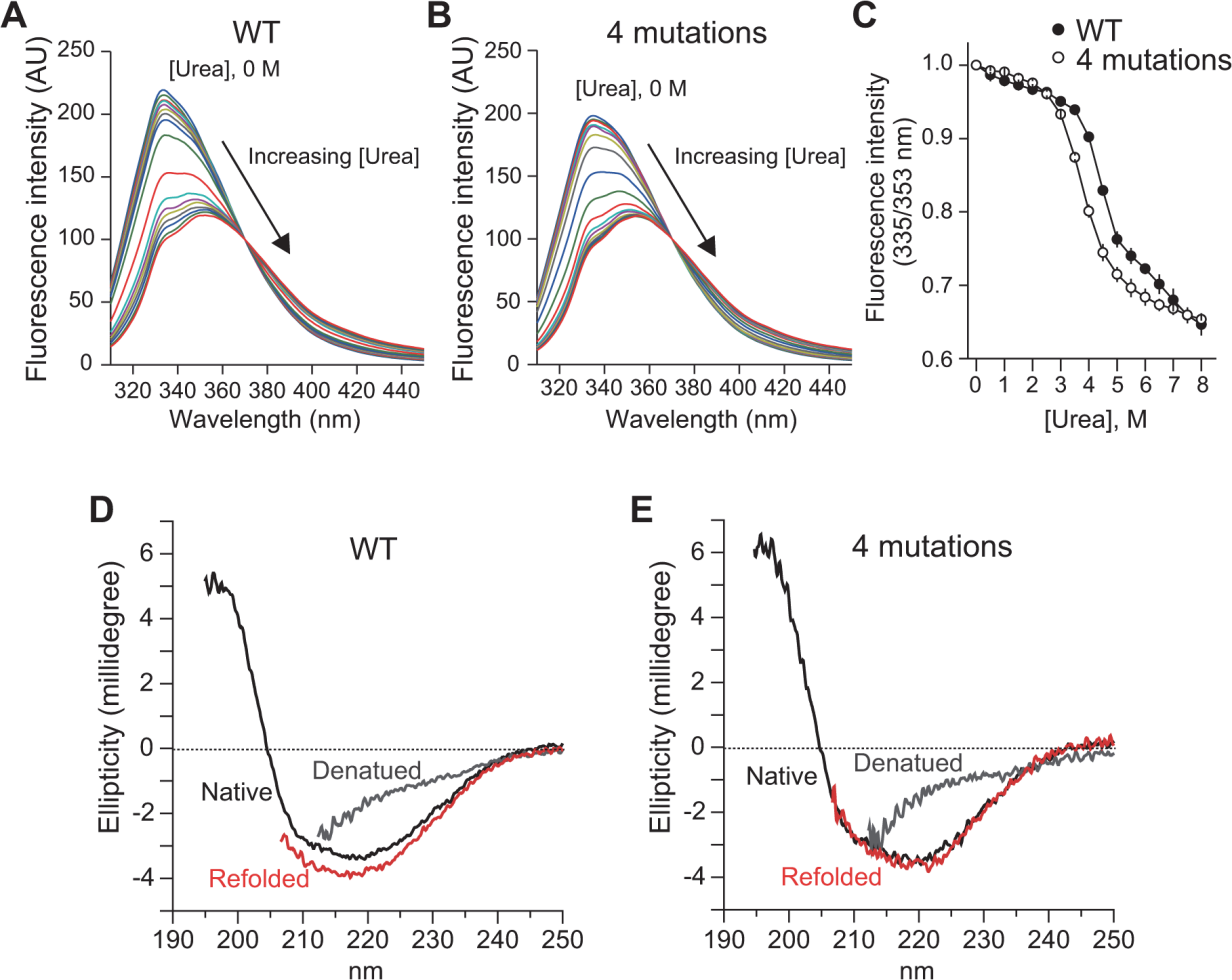
Chemical unfolding and refolding of association domain mutant. **(A, B)** Tryptophan fluorescence spectra of association domain (A) and its mutant (F394L/I419V/A430T/I434T) (B) in increasing urea concentrations (0–8 M with 0.5 M intervals) were recorded at 295 nm. **(C)** Equilibrium unfolding transitions defined as the ratio of fluorescence intensities at 335 nm and 353 nm are plotted as a function of denaturant concentration. Error bars indicate S.E.M. for three independent experiments. **(D, E)** Refolding of denatured association domain mutant was recorded by far-UV circular dichroism (CD) spectra. Native proteins (black) were denatured in 8 M urea and 1 mM DTT (gray), and refolded by 5-fold dilution (red). The lower spectral regions monitored with over 400 V of the photomultiplier tube voltage were cut out from respective spectra.

Since improved sfGFP folding is expected to reflect the folding efficiency of the association domain [21], we measured the tolerance of association domain mutants for denaturing conditions and their refolding efficiency (Fig. 3). To measure their tolerance of the denaturing reagent, the unfolding of purified association domain mutants was monitored by tryptophan fluorescence in the presence of various concentrations of urea (Fig. 3A-C). A concentration of up to 3 M urea resulted in slight change in the wavelength maxima. Beyond 3 M urea, the fluorescence of wild-type and mutant association domains started decreasing at 3.5 and 3 M urea with a concomitant red shift in spectra, respectively, suggesting that the mutant association domain has slight lower tolerance to urea (Fig. 3C). With further addition of urea (>5 M), the emission peak shifted towards 353 nm with a further decrease (up to 65%) in intensity because of the presence of an unfolded structure with a tryptophan in the association domain exposed to hydrophilic conditions (Fig. 3C). Next, to test if the refolding efficiency of the association domain mutant is improved compared with the wild-type, we measured the reversibility of the urea-induced unfolding using far-UV CD spectra measurement (Fig. 3D, E). The purified association domain was denatured and unfolded by reducing the urea concentration. The spectra of unfolding and refolding states were similar for the wild-type and mutant, suggesting that the contents of α-helix and β-sheet are similar. (Fig. 3D, E). Since the mutant association domain has slight lower tolerance to urea compared with wild-type (Fig. 3A-C) and similar refolding characteristics (Fig. 3D, E), improved sfGFP folding may not be due to the improved folding of the association domain mutant, but rather the reduced interaction of the mutant association domain with the fluorescent protein in the intermediate state.

**Fig 4:**
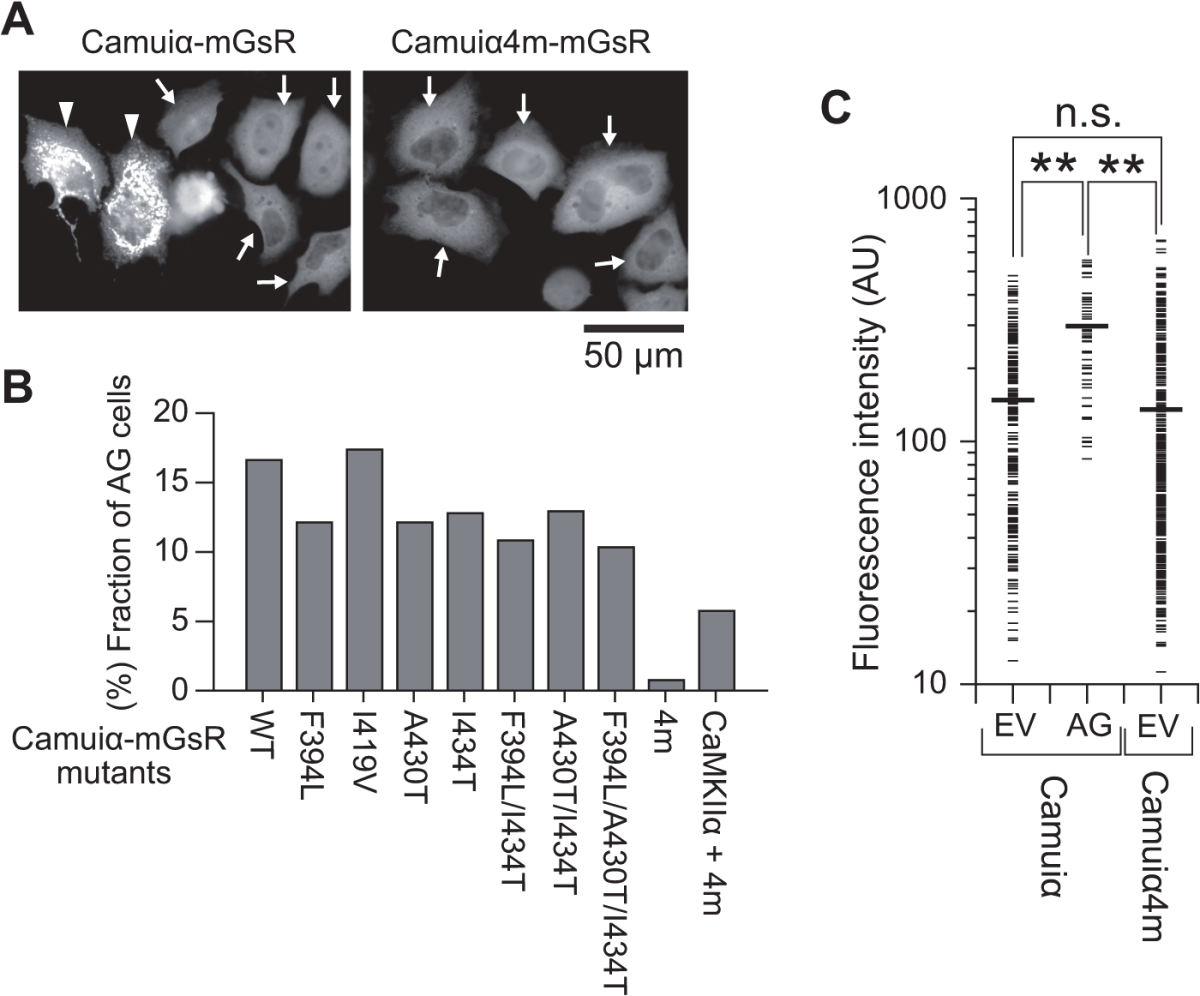
Expression patterns of the Camuiα-mGsR mutants in HeLa cells. **(A)** Camuiα mutants were expressed in HeLa cells and imaged under an epifluorescence microscope. Scale bar = 50 μm. The arrowheads highlight cells that exhibit aggregation of the FRET sensor in a dotted pattern. The arrows indicate the cells evenly expressing the Camuiα mutant. **(B)** The number of cells with aggregated Camuiα mutants in the dotted pattern were counted (wild-type, 16.6% (141/848 cells); F394L, 12.1% (97/800 cells); I419V, 17.4% (139/800 cells); A430T, 12.1% (87/717 cells); I434T, 12.8% (108/844 cells); F394L/I434T, 10.8% (81/748 cells); A430T/I434T, 12.9% (110/850 cells); F394L/A430T/I434T, 10.3% (79/765 cells); Camuiα4m, 0.8% (8/1038 cells)). **(C)** The fluorescence intensity distributions of the individual cells expressing Camuiα-mGsR and Camuiα4m-mGsR. The cells evenly expressing Camuiα mutant are designated EV cells, and the cells exhibiting aggregated Camuiα mutant are designated AG cells. Thick black horizontal bars show average intensities. The number of the cells analyzed (Camuiα (EV), Camuiα (AG), Camuiα4m) are 250, 62, and 401, respectively. For the statistical test, one-way ANOVA with Scheffé’s post-hoc test with *p < 0.05 was used. The cell exhibiting the aggregated form of Camuiα4m was not observed in this experiment.

**Fig 5:**
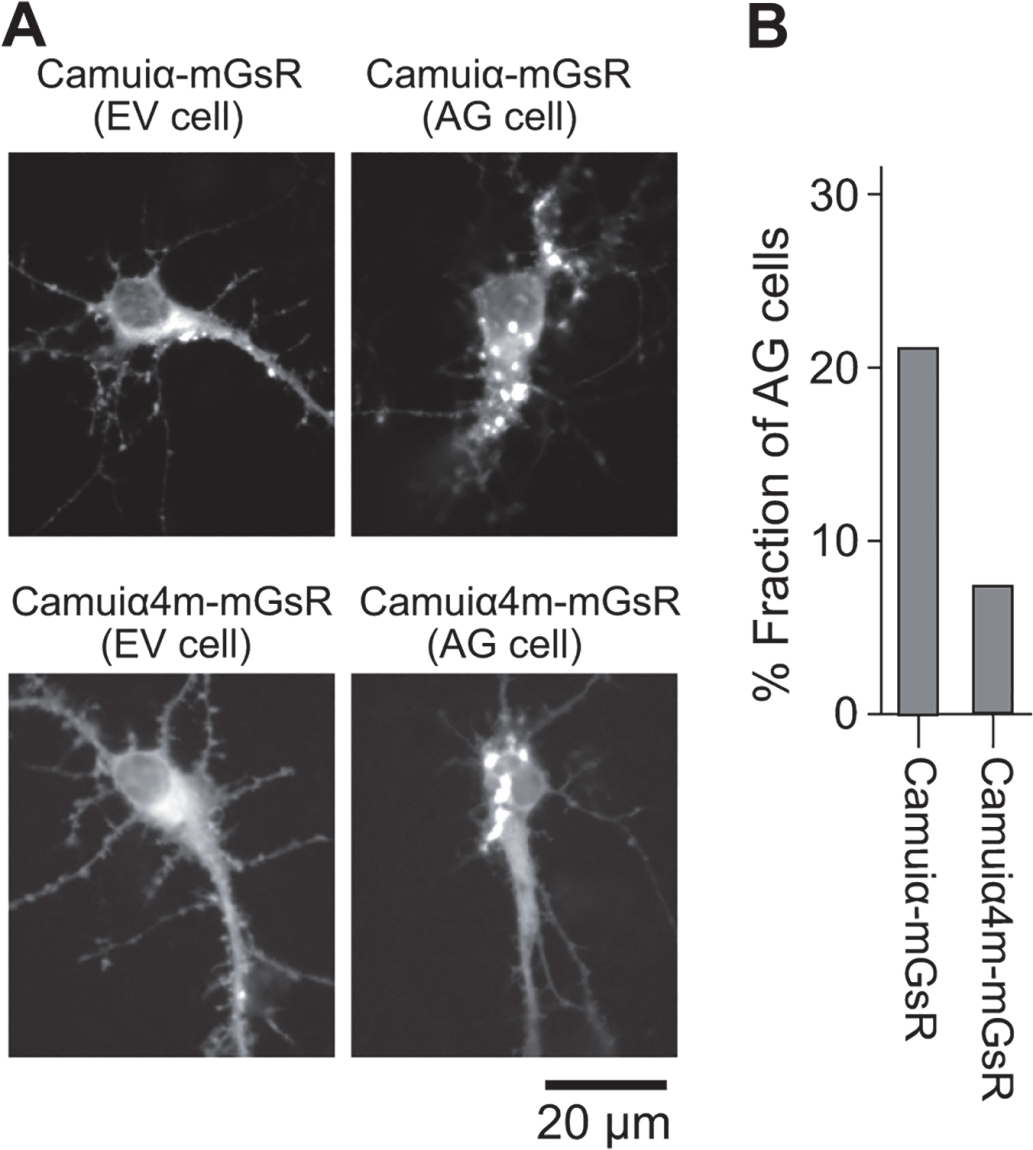
Expression patterns of the Camuiα-mGsR mutants in dissociated hippocampal neurons. **(A)** Hippocampal neurons were cultured, transfected, and imaged as described in the Methods. Neurons exhibited evenly distributed (left, EV cell) and aggregated (right, AG cell) Camuiα-mGsR (Top) and Camuiα4m-mGsR (Bottom). Scale bar = 20 μm. **(B)** The number of neurons with aggregated Camuiα in soma were counted for the Camuiα mutants (Camuiα, 21.3% (23/108 cells); Camuiα4m, 7.4% (8/108 cells)).

To test the effects of the identified mutations for the expression pattern of Camuiα, we introduced these mutations into mEGFP and sREACh-based Camuiα, Camuiα-mGsR, and then expressed the sensors in HeLa cells (Fig. 4). Interestingly, HeLa cells expressing Camuiα-mGsR consisted of two populations: one that exhibited aggregated Camuiα-mGsR fluorescence signals in a dotted pattern (designated AG cells), and another that exhibited evenly distributed Camuiα-mGsR fluorescence (Fig. 4A). The fraction of cells, expressing wild-type Camuiα-mGsR that were determined to be AG cells, was approximately 16.6%, which is not a negligible fraction (Fig. 4B). Similar results were observed for the mutants with single or double mutations (Fig. 4B). Notably, the aggregation of Camuiα-mGsR did not localize to the mitochondria, endoplasmic reticulum, Golgi body, or lysosome (data not shown). Further, the introduction of three mutations (F394L, A430T, and I434T) in the association domain slightly reduced the population of AG cells to 10.3% compared with the wild-type; this is still a large fraction. However, the introduction of all four mutations (F394L, I419V, A430T, and I434T) dramatically reduced the fraction of AG cells to 0.8%, improving expression pattern. Therefore, we have named this new Camuiα construct, Camuiα4m-mGsR. The improved expression pattern of Camuiα4m-mGsR was also observed in HeLa cells co-expressing CaMKIIα (Fig. 4B) and hippocampal neurons endogenously expressing CaMKIIα (Fig. 5). One possible explanation for the improved expression pattern of Camuiα4m-mGsR is the reduced expression level of Camuiα4m-mGsR, since AG cells tend to exhibit a high level of expression of Camuiα-mGsR (Fig. 4C). However, the similarly high expression of Camuiα4m-mGsR did not show aggregation (Fig. 4C).

**Fig 6:**
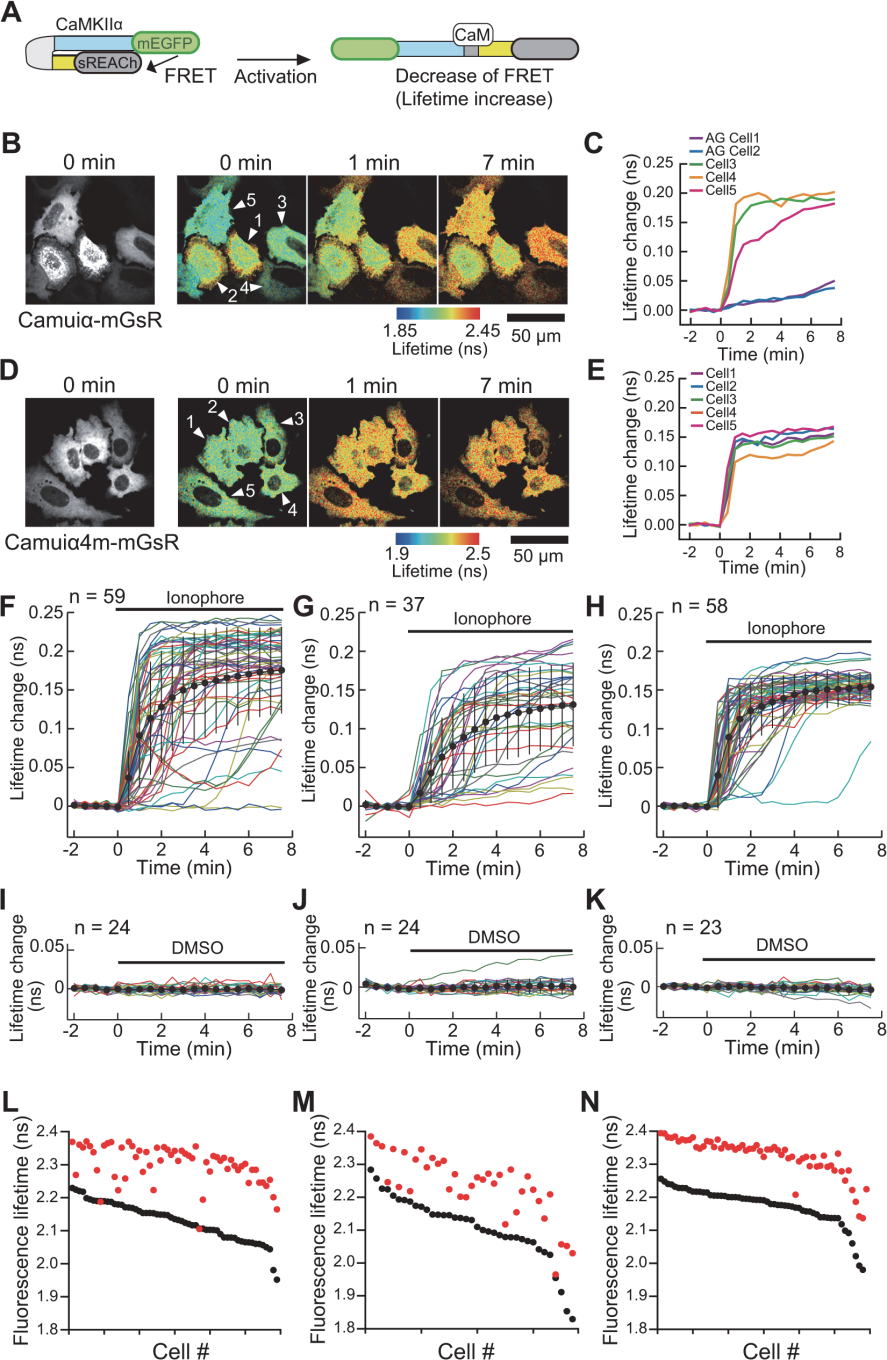
Characterization of Camuiα4m-mGsR mutant activation in living cells. **(A)** Schematic drawing of Camuiα activation. When Camuiα is in an inactive state, it is in the compact conformation. The binding of Ca^2+^/calmodulin induces a structural change, leading to activation. Since the structural change is associated with activity, FRET measurement can be used to monitor the activity. **(B, D)** Representative 2-photon fluorescence (left) and 2pFLIM (right) images of Camuiα-mGsR **(B)** and Camuiα4m-mGsR **(D)** co-expressed with calmodulin (DNA ratio of 1:1) in HeLa cells. The 0-minute time point indicates when 10 μM 4-bromo-A23187 ionophore stimulation was applied. Two-photon excitation at 920 nm was used to excite mEGFP in the Camuiα mutant. Scale bar = 50 μm. **(C, E)** The time course of Camuiα and Camuiα4m activity in the individual cells shown in **B** and **D**, respectively. Note that cells with aggregated sensor (AG cells) show only slight activation (e.g., cells 1 and 2 in **B** and **C**). **(F–K)** The activation of evenly expressing Camuiα in HeLa cells following ionophore (**F**) or DMSO (**I**) application. The activation of Camuiα in AG cells after ionophore (**G**) or DMSO (**J**) application. The activation of Camuiα4m after ionophore (**H**) or DMSO (**K**) application. Colored lines represent the response signals from individual cells and the black circles indicate an averaged time course. Error bars represent mean ± SD. The numbers of cells (n) is indicated in the Fig. **(L–N)** The basal fluorescence lifetimes (average of -2 to 0 minutes) of individual cells are plotted in descending order (black) along with the corresponding fluorescence lifetimes (average of 5.5 to 7.5 minutes) after ionophore stimulation (red). The data from **(F)**, **(G)**, and **(H)** are used in **(L)**, **(M)**, and **(N)**, respectively.

**Fig 7:**
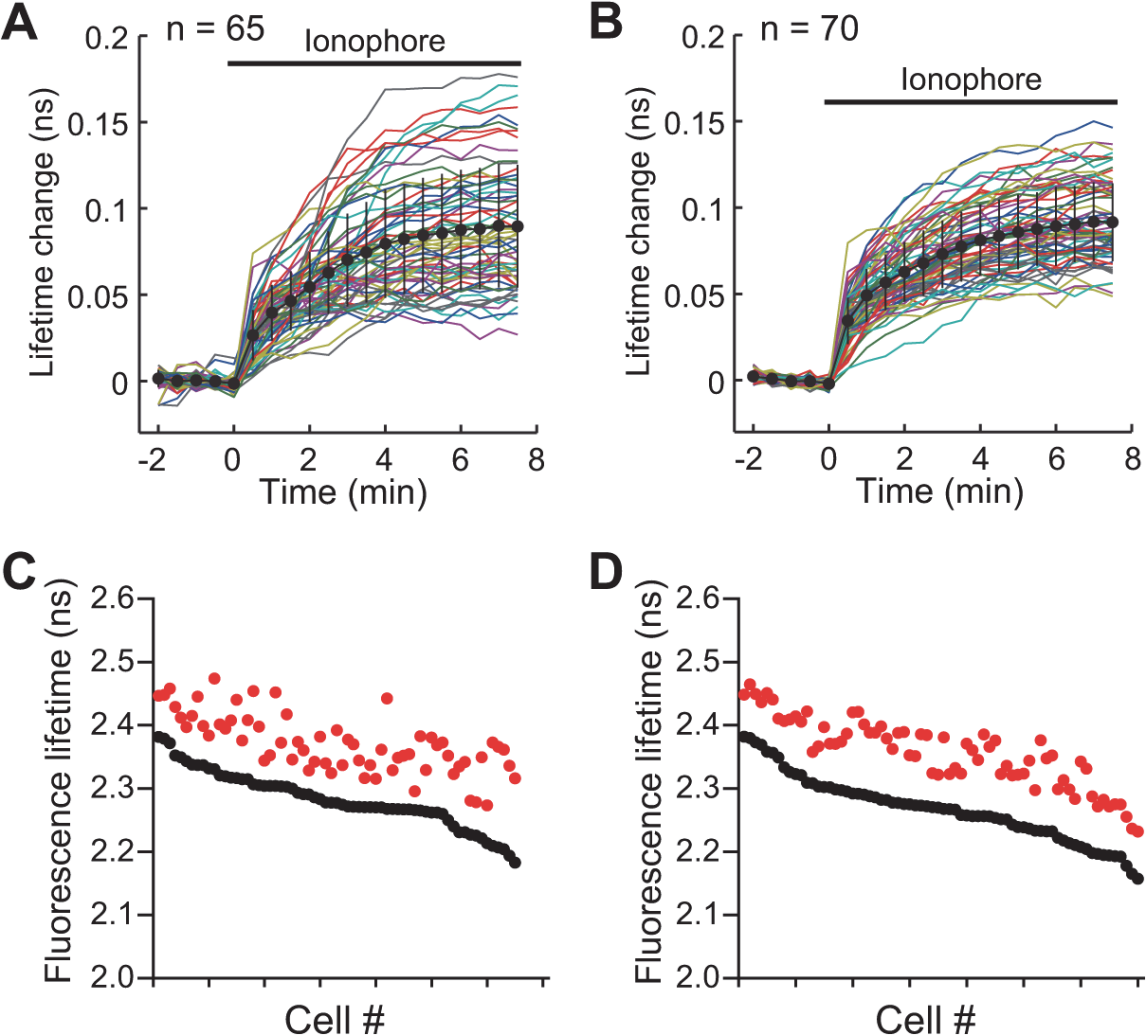
Characterization of Camuiα4m-mGsR mutant activation with co-expression of CaMKIIα. **(A, B)** The time course of Camuiα (A) and Camuiα4m (B) activity in the individual HeLa cells. The cells expressing Camuiα-mGsR or Camuiα4m-mGsR with non-labeled CaMKIIα and calmodulin (DNA ratio of 1:2:3) were stimulated with 10 μM 4-bromo-A23187 ionophore and monitored by 2pFLIM. **(C, D)** The basal fluorescence lifetimes (average of -2 to 0 minutes) of individual cells are plotted in descending order (black) along with the corresponding fluorescence lifetimes (average of 5.5 to 7.5 minutes) after ionophore stimulation (red). The data from **(A)** and **(B)** are used in **(C)** and **(D)**, respectively.

Next, to evaluate the sensitivity of the sensor, we compared the FRET signals of Camuiα4m-mGsR and Camuiα-mGsR in HeLa cells using 2pFLIM [22,29]. In this experiment, Camuiα4m-mGsR or Camuiα-mGsR was co-transfected with calmodulin that is not expressed at high enough levels in HeLa cells, but is required for CaMKII activation. First, Camuiα-mGsR activation in individual cells was monitored (Fig. 6). Because HeLa cells expressing Camuiα-mGsR comprised two populations—AG cells and the cells evenly expressing the sensor, as mentioned in the previous section—we analyzed cells from each population separately (Fig. 6F, G). The cells that exhibited uniform expression showed robust activation after ionophore stimulation (cells 3, 4, and 5 in Fig. 6C, F), whereas the AG cells showed significantly decreased levels of response signal, most likely owing to the unfavorable aggregation of Camuiα-mGsR (cells 1 and 2 in Fig. 6C, G). Next, we measured the FRET signal of Camuiα4m-mGsR after ionophore stimulation (Fig. 6H), and compared these results with the wild-type cells evenly expressing Camuiα-mGsR (Fig. 6F). Interestingly, the cells expressing Camuiα4m-mGsR showed robust FRET signals with remarkably minimized standard deviation (mean ± SD at 7 minutes was 0.153 ± 0.016, Fig. 6H), compared with Camuiα-mGsR (mean ± SD at 7 minutes was 0.174 ± 0.056, Fig. 6F). These data indicate that Camuiα4m-mGsR has a more stable response to the stimulation compared with that of Camuiα-mGsR (Fig. 6F, H). No activation was observed in either cell population when DMSO was added (Fig. 6I, J, K), suggesting that the activation is due to the Ca^2+^ influx induced by the ionophore. The decreased response variability of cells expressing Camui4mα-mGsR is due to the smaller fluorescence lifetime variability in both before and after stimulation (Fig. 6L-N). The similar results were obtained from the experiments with co-expression of CaMKIIα (Fig. 7). Unexpectedly, the average lifetime change is slightly larger for Camuiα-mGsR (0.174 ns) compared with Camuiα4m-mGsR (0.153 ns), which is due to the smaller basal fluorescence lifetime of Camuiα-mGsR (Fig. 6L, N).

**Fig 8:**
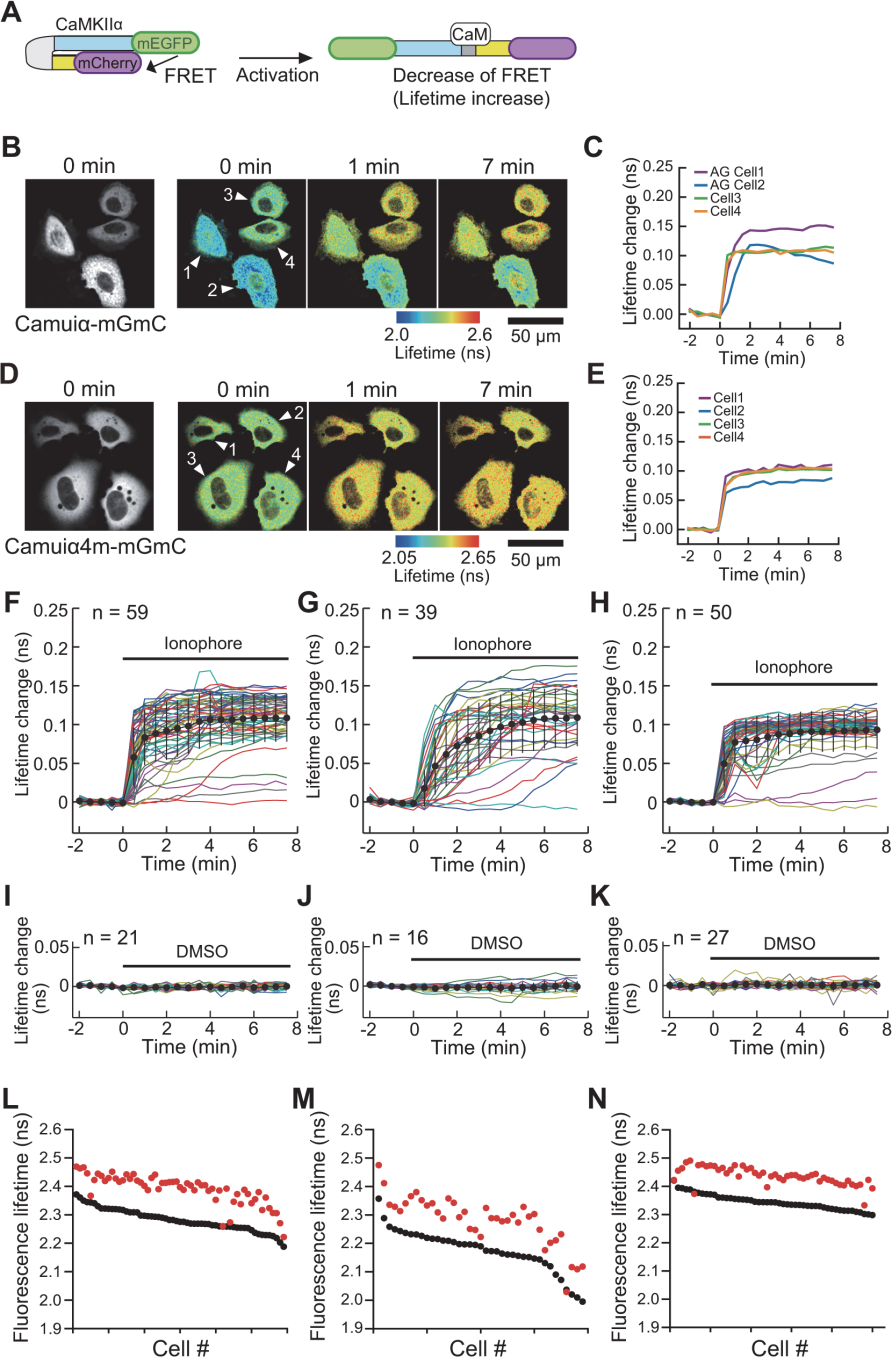
Characterization of Camuiα4m-mGmC mutant activation in living cells. The activities of mEGFP- and mCherry-based Camuiα mutants (Camuiα-mGmC, Camuiα4m-mGmC) were monitored. The experimental conditions, analyses, and Fig. captions are the same as in Fig. 6, except for the replacement of sREACh by mCherry in Camuiα mutants.

We also carried out the same set of experiments with mCherry-based Camuiα mutants, and found that the response variability of Camuiα4m-mGmC was smaller than that of Camuiα-mGmC, similar to the results for the sREACh-based Camuiα (Fig. 8). However, the sREACh version of Camuiα showed a greater response signal compared to the mCherry version (Figs. 6H, 8H), consistent with the previously reported results showing the greater maturation efficiency of sREACh compared with mCherry [22].

**Fig 9:**
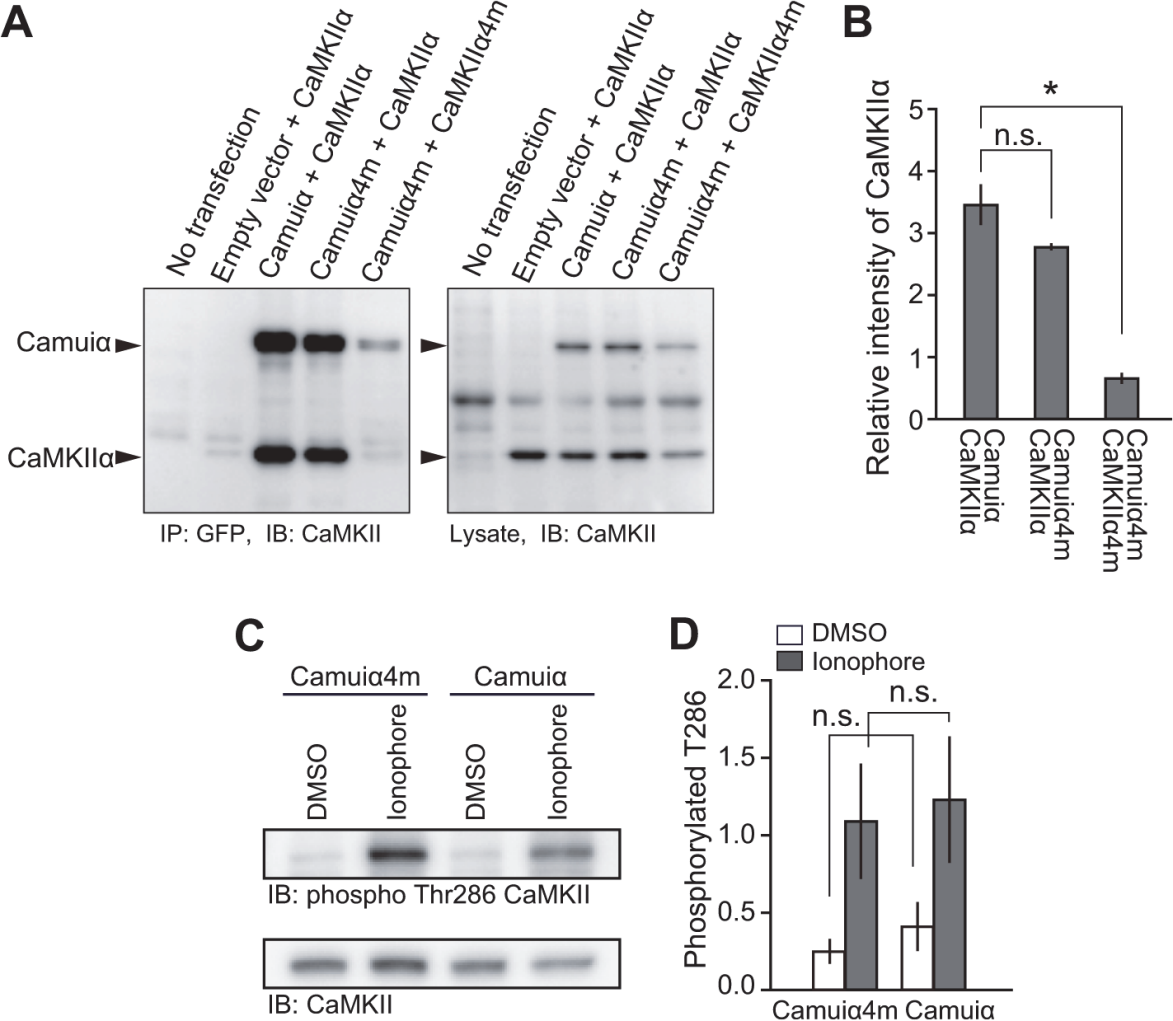
Immunoprecipitation and kinase activity analyses. **(A)** HeLa cells expressing Camuiα-mGsR or Camuiα4m-mGsR with non-labeled CaMKIIα or CaMKIIα4m (DNA ratio of 1:2) were lysed and immunoprecipitated with anti-GFP antibody followed by immunoblotting with CaMKII antibody. **(B)** Quantitative analysis of immunoprecipitation. The band intensities of immunoprecipitated Camuiα and its mutant were divided by those of the lysates. Error bars indicate S.E.M. for three independent experiments. For the statistical test, one-way ANOVA with Scheffé’s post-hoc test with *p < 0.05 was used. **(C)** HeLa cells expressing Camuiα mutants and calmodulin (DNA ratio of 1:1) were stimulated with 20 μM of 4-bromo-A23187 ionophore for 1 or 2 minutes. Subsequently, the cells were lysed and immunoblotted with anti-phosphoThr286 CaMKII antibody. As a control experiment, DMSO was added instead of ionophore. **(D)** Quantitative analysis of Camuiα mutant activation. The band intensities of phosphorylated Camuiα mutant were divided by those of the lysates. Error bars indicate S.E.M. for three independent experiments. There were no significant differences between the phosphorylation levels of Camuiα and Camuiα4m (p > 0.05, *t*-test, n.s., not significant).

**Fig 10:**
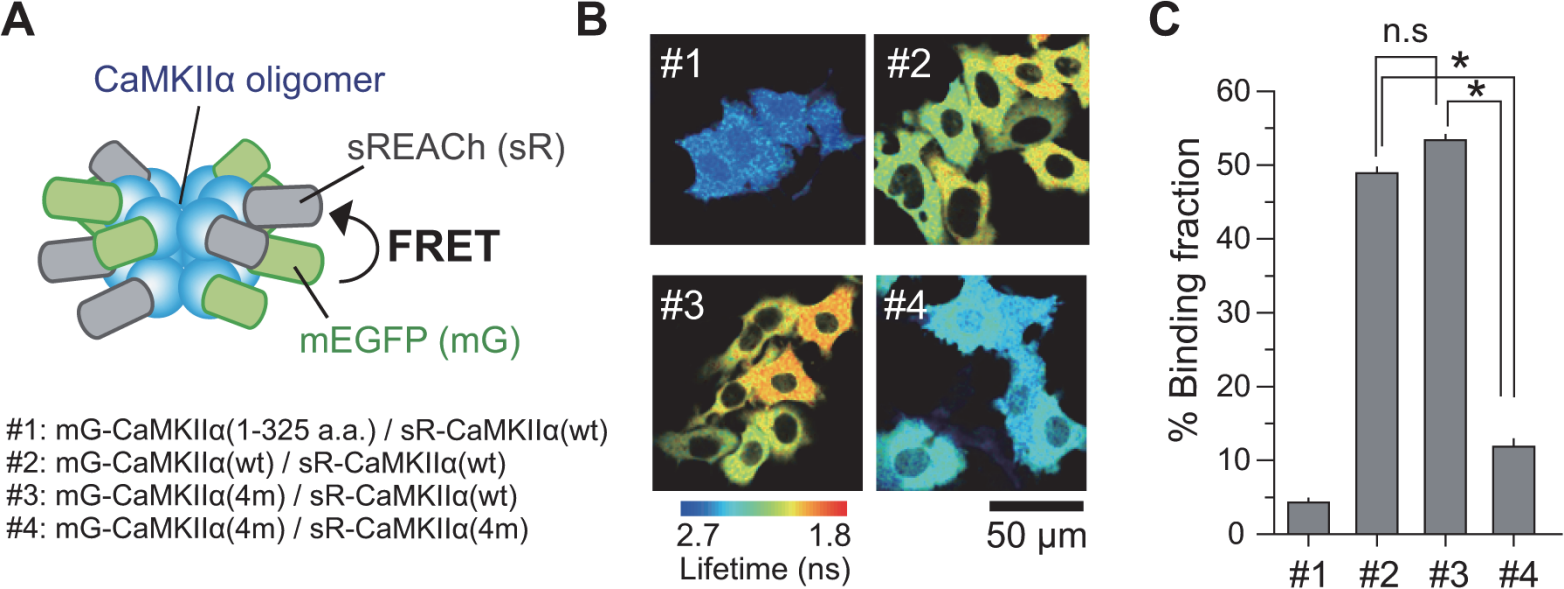
Oligomerization status of CaMKIIα mutants in living cells. **(A)** Schematic drawing of the experimental design. Inter-molecular FRET between mEGFP-fused CaMKIIα (or CaMKIIα4m mutant) and sREACh-fused CaMKIIα (or CaMKIIα4m mutant) were monitored by 2-photon fluorescence lifetime microscope. mEGFP-CaMKIIα (1–325 a.a.) lacking association domain was used as negative control. **(B)** Representative fluorescence lifetime images of HeLa cells expressing the pair of CaMKIIα. The ratio of FRET donor and acceptor DNA plasmids transfected was 1:2. The binding fractions were measured from fluorescence decay curves as described elsewhere [22]. Scale bar = 50 μm. **(C)** Comparison of the binding fractions. The number of the cells analyzed (#1–#4) are 83, 89, 82, and 103, respectively. Error bars indicate S.E.M. For the statistical test, one-way ANOVA with Scheffé’s post-hoc test with *p < 0.05 was used.

**Fig 11:**
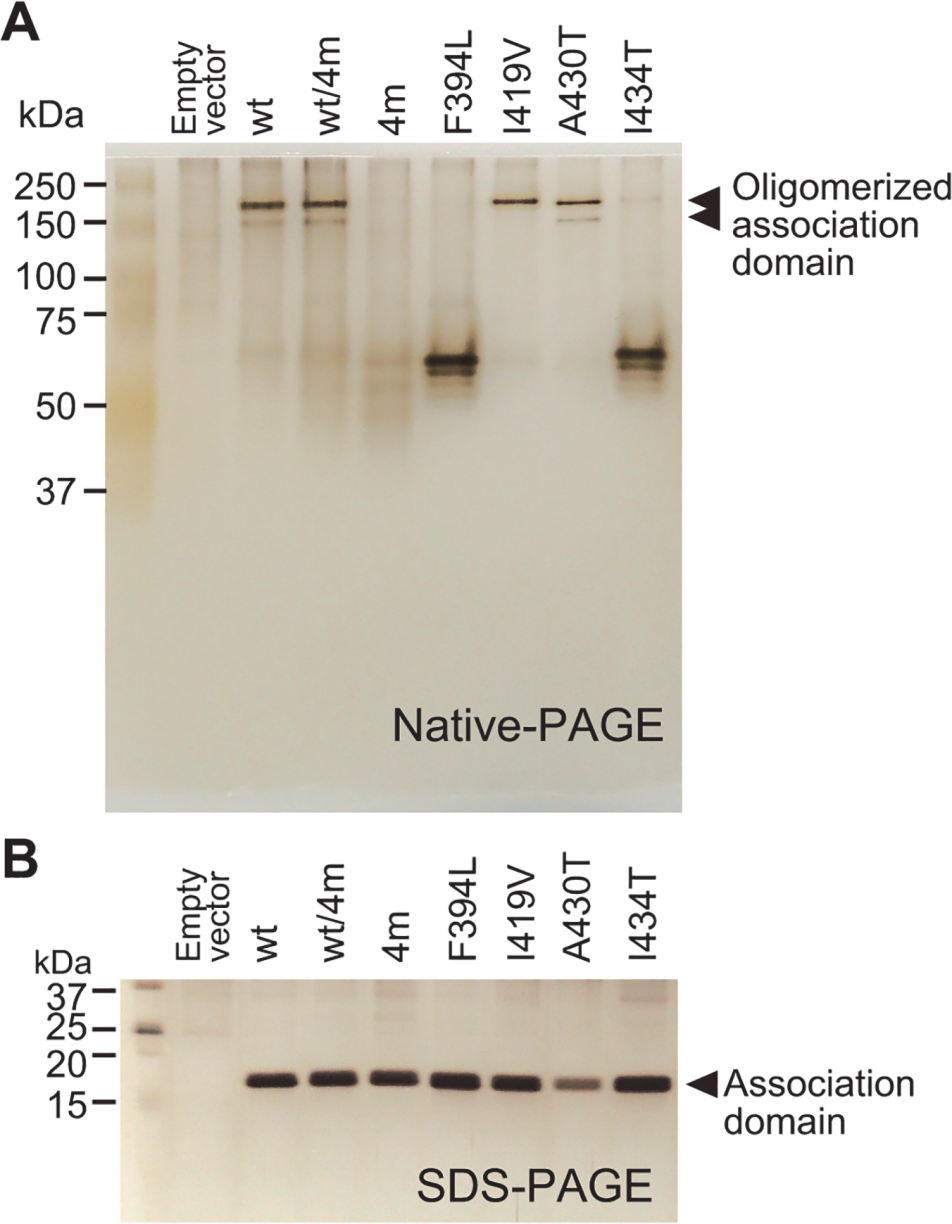
Native-PAGE analysis of association domain mutants. **(A)** His-tagged association domain mutants (molecular weight; 19,800) expressed in HeLa cells were purified with Ni-sepharose resin as described in Materials and Methods. Subsequently, native-PAGE and silver staining were carried out. The two arrowheads indicate oligomer forms of the association domain. **(B)** SDS-PAGE of purified association domain mutants. The same batch of samples as in (A) was used.

Among the identified four mutations, F394L, A430T, and I434T face the binding regions of an adjacent association domain (Fig. 1B) [36]. This fact raises the possibility that these mutations may inhibit proper association between the CaMKII subunits. Therefore, to test if oligomerization was hindered, we co-transfected HeLa cells with Camuiα4m-mGsR and CaMKIIα and immunoprecipitated the proteins using an anti-GFP antibody. Western blotting revealed the presence of two bands corresponding to the molecular weights of Camuiα4m-mGsR and CaMKIIα (Fig. 9). In addition, the ratio of the band intensities of Camuiα4m-mGsR and CaMKIIα was similar to that of Camuiα-mGsR and CaMKIIα, suggesting that Camuiα4m-mGsR is incorporated into CaMKIIα in a similar way to Camuiα. However, when CaMKIIα4m and Camuiα4m-mGsR were co-expressed and immunoprecipitated, the interaction between CaMKIIα4m and Camuiα4m-mGsR was dramatically decreased, suggesting that the association domain with four mutations does not form a homo-oligomer (Fig. 9A, B). Consistent results were obtained by monitoring the inter-molecular FRET in living HeLa cells using 2pFLIM (Fig. 10). These facts could explain that the reason for the smaller basal fluorescence lifetime of Camuiα in Figs. 6 and 8 may be due to the inter-molecular FRET between Camuiα sensors, further increasing basal FRET, because Camuiα association domain forms tight oligomer compared with association domain with four mutations (Figs. 9, 10). Next, to identify the mutation that hinders the oligomeric formation, we carried out native-PAGE analysis and found that F394L and A434T hinder oligomer formation (Fig. 11). Furthermore, we tested the Ca^2+^/calmodulin-dependent autophosphorylation capabilities of Camuiα4m-mGsR in HeLa cells by western blotting with anti-phospho antibody. After stimulation of the cells with ionophore, Camuiα4m was shown to autophosphorylate in a similar manner to the wild-type Camuiα-mGsR (Fig. 9C, D), suggesting that Camuiα4m-mGsR is activated in a similar way.

**Fig 12:**
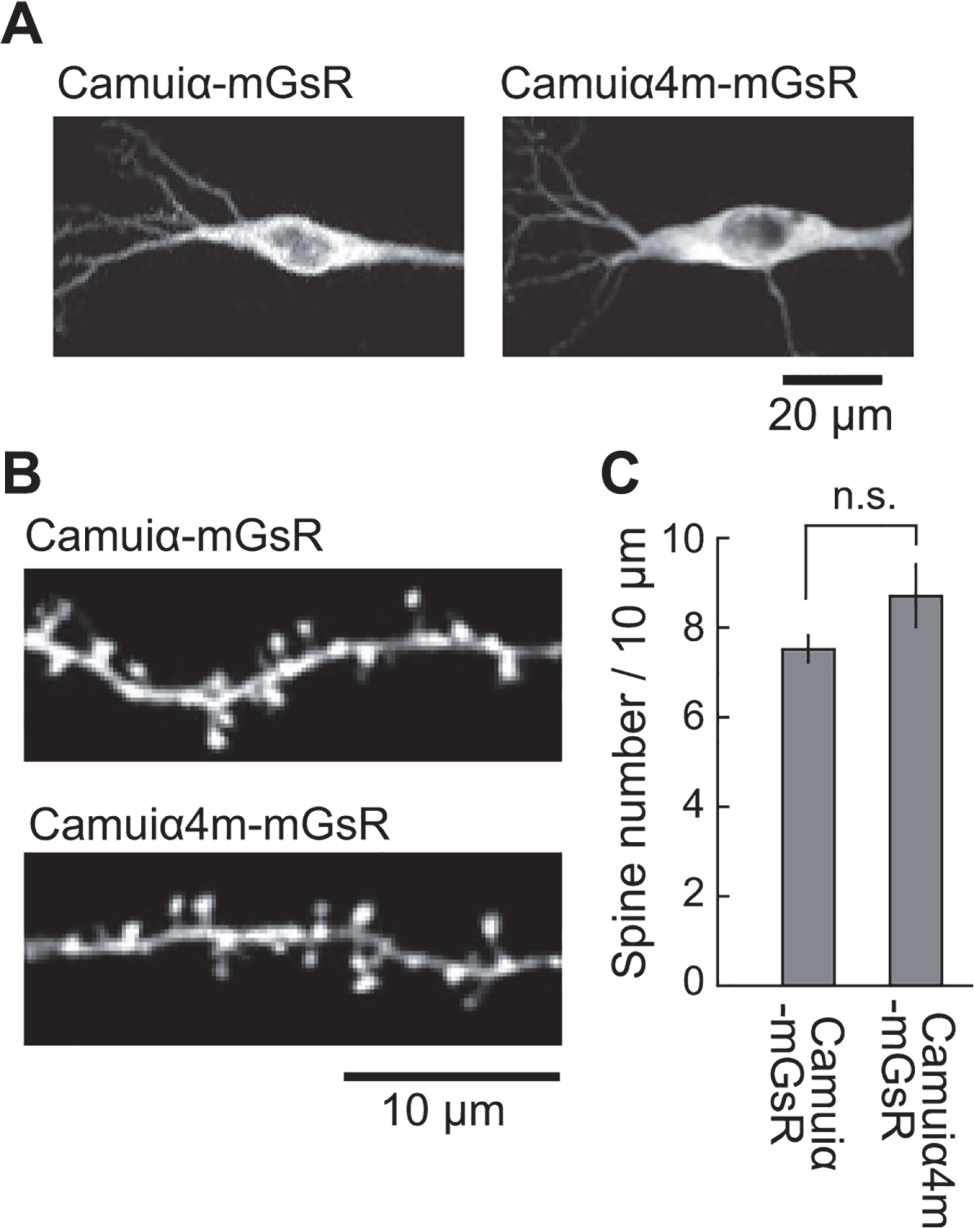
Two-photon fluorescence images of the neurons expressing Camuiα4 mutants in hippocampal slice culture. **(A)** Representative images of soma of the CA1 pyramidal neuron expressing Camuiα-mGsR or Camuiα4m-mGsR. Either Camuiα-mGsR or Camuiα4m-mGsR was transfected using a gene gun, and 2 days after transfection, cells were imaged with a 2-photon fluorescence microscope. Scale bar = 20 μm. **(B)** Representative images of apical dendrites from CA1 pyramidal neurons expressing Camuiα-mGsR or Camuiα4m-mGsR. Scale bar = 10 μm. **(C)** Spine density in apical dendrites of neurons. The numbers of samples (spines/neurons) are 1541/7 for Camuiα-mGsR and 1827/7 for Camuiα4m-mGsR. There were no significant differences between the spine density in neurons expressing Camuiα and Camuiα4m (p > 0.05, *t*-test, n.s., not significant). Error bars indicate S.E.M.

**Fig 13:**
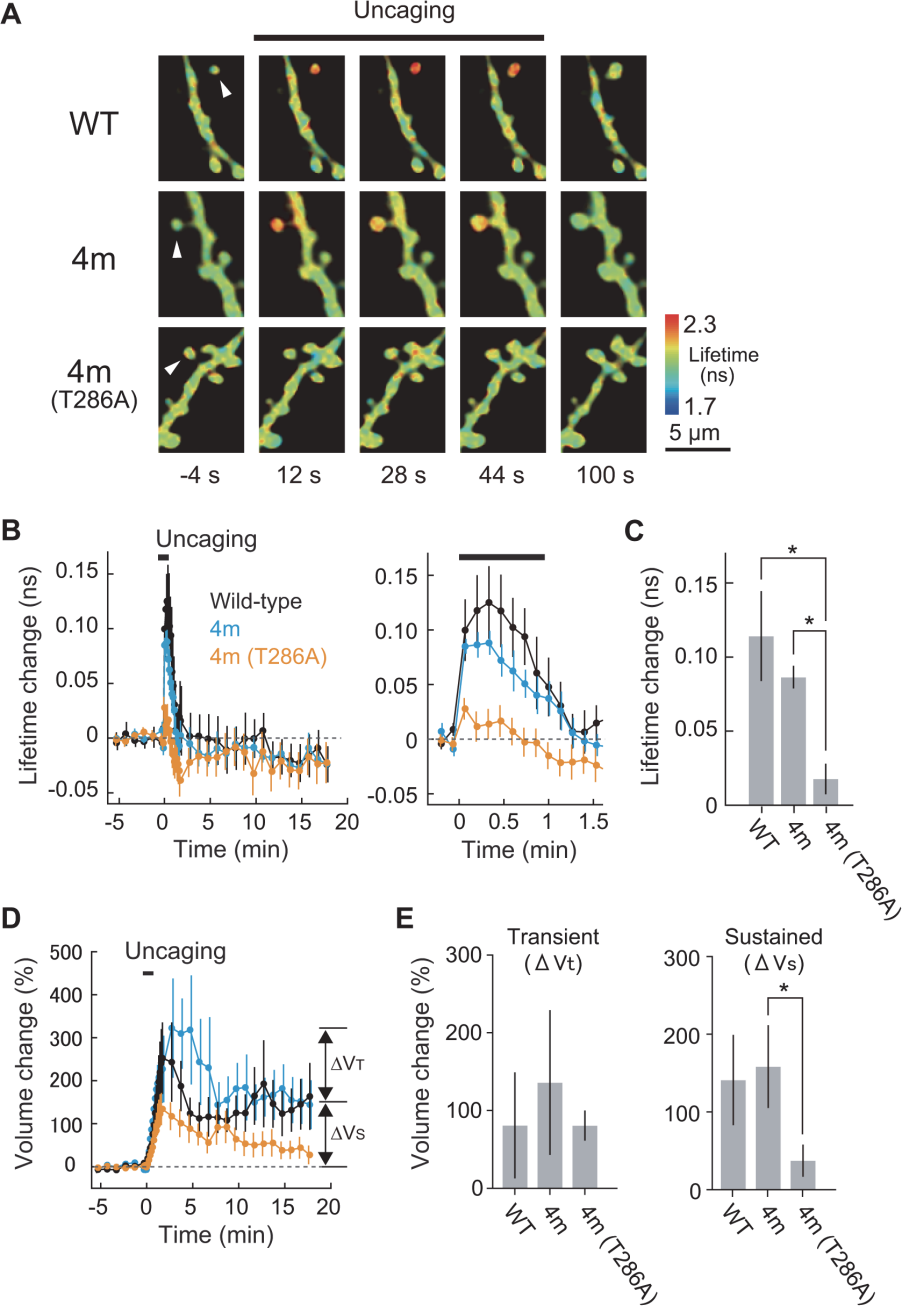
Camuiα4m-mGsR activation by single-spine stimulation. **(A)** Fluorescence lifetime images of neurons expressing Camuiα4m-mGsR together with tandem mCherry during the induction of spine enlargement by 2-photon glutamate uncaging. The arrowhead indicates the stimulated spine. **(B)** Time courses of Camuiα mutants activities measured as a change in the fluorescence lifetime of Camuiα-mGsR, Camuiα4m-mGsR, Camuiα4m (T286A)-mGsR in the stimulated spines are overlaid with the spine volume change measured as a change in the mCherry fluorescence, respectively. The numbers of samples (spines/neurons) are 6/4 for wild-type, 9/5 for 4m, and 10/6 for 4m with T286A, respectively. Error bars are S.E.M. in **B**-**E**. **(C)** Peak Camuiα mutants activation (averaged over 0–24 s). Stars denote statistical significance (p < 0.05, ANOVA followed by the least significant difference (LSD) test). (**D)** Averaged time courses of spine volume change in the same experiments in **B**. (**E)** Transient (volume change averaged over 2–4 min subtracted by that over 15–18 min) and sustained volume change (volume change averaged over 15–18 min). Stars denote statistical significance (p < 0.05, ANOVA followed by the LSD tests).

Since CaMKIIα is specifically expressed in neurons, Camuiα4m-mGsR will be used in neurons for monitoring CaMKIIα activity. Therefore, we tested if Camuiα4m-mGsR expression alters the dendritic spine density of hippocampal neurons, compared with Camuiα-mGsR, and found that there was no significant difference between Camuiα-mGsR and Camuiα4m-mGsR (Fig. 12), showing that there is no unfavorable effect on the neuronal morphology. Furthermore, we monitored Camuiα4m-mGsR activity during spine enlargement upon local glutamate uncaging [37], and found that both activity and spine volume changes are similar to those of Camuiα-mGsR (Fig. 13). In contrast, Camuiα4m-mGsR with T286A mutation which abolishes autophosphorylation showed the decreased activation and spine volume change (Fig. 13), consistent with the previously reported result with Camuiα [15,16]. Taken together, these results indicate that Camuiα4m-mGsR may be useful for monitoring CaMKII activity.

## Conclusions

We have demonstrated here that molecular evolution of the association domain in the Camuiα FRET sensor successfully improves its expression pattern and minimizes response variability (Figs. 4, 5, 6, 7, 8). While a significant fraction of cells expressing Camuiα-mGsR exhibited aggregation, the cells expressing Camuiα4m-mGsR did not (Fig. 4). The reason for the improved expression may be owing to the improved folding kinetics of the downstream fluorescent protein (Fig. 2), not owing to the improved folding of the association domain itself (Fig. 3). Unexpectedly, the introduction of four mutations (F394L, I419V, A430T, and I434T) hinders homo-oligomer formation, but not hetero-oligomer formation between wild-type and mutant association domains (Figs. 9, 10, 11). One possible explanation for the improved expression pattern of Camuiα4m-mGsR is its inability to form oligomers. However, since the introduction of a single mutation, namely F394L or I434T, disrupts oligomerization but does not improve the expression pattern (Figs. 4, 11), the inability to form oligomers may not be the reason for the improved expression pattern. Thus, we speculate that the decreased large aggregation of Camuiα4m is due to the decreased non-specific binding between inter-association domains and between association domain and GFP, sREACh, and mCherry in the folding-intermediate state [35].

FRET experiments confirmed that Camuiα4m-mGsR exhibits a more robust response with smaller response variability than Camuiα-mGsR. This minimized response is probably due to the minimized variability of both the basal fluorescence lifetime and the response signal (Figs. 6, 8). Another potentially important reason for the decrease in variability may be an effect of the increased folding efficiency of the C-terminal fluorescent protein in Camuiα4m mutant (Fig. 2). Since the folding efficiency of sfGFP fused to the C-terminus of the association domain was greatly improved in terms of refolding rate and correctly refolded fraction (Fig. 2, Table 1), the folding efficiency of the C-terminal fluorescent proteins, sREACh and mCherry, may also be improved and could contribute to the minimized cell-to-cell variability of fluorescence lifetime. The Camuiα4m-mGsR developed here exhibits a robust response and can be utilized to measure the CaMKIIα activity more precisely *in vitro* and *in vivo*. Furthermore, the directed mutagenesis strategy used here may be useful for improving the quality of all FRET sensors in terms of response variability.
